# Supplementary material for: Content-rich biological network constructed by mining PubMed abstracts
Source: BMC Bioinformatics. 2004 Oct 8;5:147. doi: 10.1186/1471-2105-5-147 (PMC528731; doi:10.1186/1471-2105-5-147)
Supplement: Additional File 5 — The original Chilibot query results of the term "long-term potentiation (LTP)" and 22 other terms, limiting the latest references analyzed to the years 1990, 1995, 2000, and 2004. [file 1471-2105-5-147-S5.bz2 › chilibotAdditionalFile5/ltp2004/html/LTP_KV4.2.html]

 


 **LTP** and **KV4.2** 
  
Found 1 abstracts in PubMed,  **1 abstracts were retrieved and analyzed**.  


---

 Search Google  |
 PDF files only 
|  EDU domain only 

---

**Interactive relationship** (e.g. stimulation, inhibition, etc)

- This expression selectively decreased basal phosphorylation of a membrane associated pool of p42 44MAPK, impaired cAMP dependent  **LTP**  in the hippocampal Schaffer collateral pathway induced by either forskolin or theta frequency stimulation, decreased complex spike firing, and reduced the p42 44MAPK mediated phosphorylation of the A type potassium channel  **Kv4.2** .  Ref: 12873387 Neuron, 2003

- :-)
